# The *UGT1A6*_19_GG genotype is a breast cancer risk factor

**DOI:** 10.3389/fgene.2013.00104

**Published:** 2013-06-11

**Authors:** Christina Justenhoven, Ofure Obazee, Stefan Winter, Sylvia Rabstein, Anne Lotz, Volker Harth, Beate Pesch, Thomas Brüning, Christian Baisch, Jaana M. Hartikainen, Arto Mannermaa, Veli-Matti Kosma, Vesa Kataja, Robert Winqvist, Katri Pylkäs, Arja Jukkola-Vuorinen, Mervi Grip, Peter A. Fasching, Matthias Beckmann, Arif B. Ekici, Alexander Hein, Per Hall, Jingmei Li, Jenny Chang-Claude, Dieter Flesch-Janys, Petra Seibold, Anja Rudolph, Ute Hamann, Yon-Dschun Ko, Hiltrud Brauch

**Affiliations:** ^1^Dr. Margarete Fischer-Bosch-Institute of Clinical Pharmacology, University of TuebingenStuttgart, Germany; ^2^Institute for Prevention and Occupational Medicine of the German Social Accident InsuranceBochum, Germany; ^3^Institute for Occupational Medicine and Maritime Medicine, University Medical Center Hamburg-EppendorfHamburg, Germany; ^4^Department of Internal Medicine, Evangelische Kliniken Bonn gGmbH, Johanniter KrankenhausBonn, Germany; ^5^Cancer Center of Eastern Finland, School of Medicine, Institute of Clinical Medicine, Pathology and Forensic Medicine, University of Eastern Finland, Biocenter KuopioKuopio, Finland; ^6^Department of Pathology, Imaging Center, Kuopio University HospitalKuopio, Finland; ^7^Cancer Center, Kuopio University HospitalKuopio, Finland; ^8^Cancer Center of Eastern Finland, School of Medicine, Institute of Clinical Medicine Oncology, University of Eastern FinlandBiocenter Kuopio, Kuopio, Finland; ^9^Laboratory of Cancer Genetics and Tumor Biology, Department of Clinical Genetics and Biocenter Oulu, Oulu University Hospital, University of OuluOulu, Finland; ^10^Department of Oncology, Oulu University Hospital, University of OuluOulu, Finland; ^11^Department of Surgery, Oulu University Hospital, University of OuluOulu, Finland; ^12^Institute of Human Genetics, University Hospital Erlangen, Friedrich-Alexander University Erlangen-NurembergErlangen, Germany; ^13^Division Hematology/Oncology, Department of Medicince, David Geffen School of Medicine, University of California at Los AngelesLos Angeles, CA, USA; ^14^Department of Medical Epidemiology and Biostatistics, Karolinska InstituteStockholm, Sweden; ^15^Human Genetics, Genome Institute of SingaporeSingapore, Singapore; ^16^Division of Cancer Epidemiology, German Cancer Research Center (DKFZ)Heidelberg, Germany; ^17^Department of Medical Biometry and Epidemiology, Center for Experimental Medicine, University Medical Center Hamburg-EppendorfHamburg, Germany; ^18^Molecular Genetics of Breast Cancer, Deutsches KrebsforschungszentrumHeidelberg, Germany

**Keywords:** UGT1A6, polymorphism, breast cancer risk, validation, metabolism

## Abstract

Validation of an association between the *UGT1A6*_19_T>G (rs6759892) polymorphism and overall breast cancer risk. A pilot study included two population-based case-control studies from Germany (MARIE-GENICA). An independent validation study comprised four independent breast cancer case-control studies from Finland (KBCP, OBCS), Germany (BBCC), and Sweden (SASBAC). The pooled analysis included 7418 cases and 8720 controls from all six studies. Participants were of European descent. Genotyping was done by MALDI-TOF MS and statistical analysis was performed by logistic regression adjusted for age and study. The increased overall breast cancer risk for women with the *UGT1A6*_19_GG genotype which was observed in the pilot study was confirmed in the set of four independent study collections (OR 1.13, 95% CI 1.05–1.22; *p* = 0.001). The pooled study showed a similar effect (OR 1.09, 95% CI 1.04–1.14; *p* = 0.001). The risk effect on the basis of allele frequencies was highly significant, the pooled analysis showed an OR of 1.11 (95% CI 1.06–1.16; *p* = 5.8 × 10^−6^). We confirmed the association of *UGT1A6*_19_GG with increased overall breast cancer risk and conclude that our result from a well powered multi-stage study adds a novel candidate to the panel of validated breast cancer susceptibility loci.

## Introductions

Many breast cancer genes have been identified including *BRCA1, BRCA2, TP53, ATM, CHEK2, BRIP1* and *PALB2* which are causative for hereditary breast cancers (Narod et al., 1995; Ford et al., 1998; Easton et al., 2007). Moreover, more than 20 common genetic variants have been shown to contribute to breast cancer risk; among these, the fibroblast growth factor receptor 2 (*FGFR2*) polymorphism rs2981582 as well as loci close to the cell cycle regulator Cyclin D1 (CCND1) at 11q13 are the most prominent risk loci (Easton et al., 2007; Lambrechts et al., 2012). Most of these loci have been identified by genome-wide association studies (GWAS) (Easton et al., 2007; Turnbull et al., 2010; Lambrechts et al., 2012). However, a shortcoming of this powerful approach is the lack of a sufficient coverage of polymorphisms in gene regions with high sequence similarities to other genes or pseudo genes. Genetic variations in such gene regions require an adapted primer design and amplification conditions which allow specific genotyping of the loci of interest (Justenhoven et al., 2010; The MARIE-GENICA Consortium, 2010). Therefore, polymorphic loci in homologous sequences are usually exempted from GWAS arrays, and can be analyzed in specially designed low-plex genotyping assays or small scale microarrays, e.g., the AmpliChip® CYP450 Test (Roche Diagnostic GmbH, Germany), the DMET™ Plus Solution (Affymetrix Inc. CA, USA) or the VeraCode® ADME Core Panel (Illumina, Inc. CA, USA) (Justenhoven, 2012). Sequence similarities are rather common among genes coding for phase I and II enzymes as well as transporters, for example P450 cytochrome 3A (CYP3A) genes with more than 70% homology (Filipits et al., 1999; Domanski et al., 2001), the UDP glucuronosyl transferases 1 (UGT1) genes with more than 50% homology (Gong et al., 2001) and the solute carrier organic anion transporter (SLCO) genes with more than 40% homology (Hagenbuch and Meier, 2004). It is of note, that these enzymes and transporters play an elementary role in the evolutionary-conserved detoxification system and in the homeostasis of endogenous molecules (Nebert, 1991; Goldstone et al., 2006).

In light of known effects of toxic compounds and steroid hormones on breast carcinogenesis, these enzymes and transporters are prime candidates for the investigation of their contribution to breast cancer risk (The MARIE-GENICA Consortium, 2010; Justenhoven et al., 2011). Thus, the UGTs are of particular interest because glucuronidation represents a major route of detoxification and elimination of xenobiotics including drugs and endobiotics (Bock and Kohle, 2005). *UGT1A6* is the founding member of the *UGT1* gene family and mediates the inactivation of phenolic compounds, e.g., 4-ethylphenol, acetaminophen or estradiol (Harding et al., 1988; Orzechowski et al., 1994; Raftogianis et al., 2000; Bock and Kohle, 2005). Two non-synonymous polymorphisms *UGT1A6*_19_T>G (rs6759892) and *UGT1A6*_541_A>G (rs2070959) are located in exon 1 causing amino acid exchanges from serine to alanine at position 7 and threonine to alanine at position 181, respectively [GenBank: P19224]. The variant alleles have been associated with decreased activity of the UGT1A6 enzyme (Ciotti et al., 1997). We previously reported their possible effect on breast cancer risk in two population-based case-control studies from Germany (The MARIE-GENICA Consortium, 2010). To validate the roles of *UGT1A6* polymorphisms in breast cancer risk we conducted an independent confirmatory study and a pooled analysis with more than 7000 patients and 8000 controls, and report the association between the *UGT1A6*_19_GG genotype and an increased overall breast cancer risk.

## Materials and methods

A total of 7148 breast cancer cases and 8472 controls from six independent cohorts were included in the analysis (Table 1). Study cohorts were selected on the basis of inclusion of female cases and controls from European descent, resident in the same region, unselected, invasive breast cancer cases and controls without history of breast cancer. Moreover, data on case-control status, age (age at diagnosis for cases and age at interview for controls), estrogen receptor (ER) and progesterone receptor (PR) needed to be available as well as DNA samples isolated from blood cells. Three studies were from Germany: the Bavarian Breast Cancer Cases and Controls Collection (BBCC), the Gene Environment Interactions and Breast Cancer study (GENICA), and the Mammary Carcinoma Risk Factor Investigation (MARIE). Two studies were from Finland: the Kuopio Breast Cancer Project (KBCP) and the Oulu Breast Cancer Study (OBCS). One study was from Sweden: the Singapore and Sweden Breast Cancer Study (SASBAC). All studies were approved by respective ethic committees and all participants gave written informed consent. Each individual study provided data on case-control status, age, ER and PR status as well as ethnicity. All study participants were of European descent.

**Table 1:** **Description of study design for each participating study**.

**Study (References)**	**Country**	**Case definition and ascertainment**	**Control definition and ascertainment**	**Age range of cases/controls**
BBCC: Bavarian Breast Cancer Cases and Controls (Fasching et al., 2008; Schrauder et al., 2008)	Germany	Consecutive, unselected cases with invasive breast cancer recruited at the University Breast Centre, Franconia in Northern Bavaria during 2002–2006.	Healthy women with no diagnosis of cancer aged 55 or older. Invited by a newspaper advertisement in Northern Bavaria, and recruited during 2002–2006.	22–96/18–100
GENICA: Gene Environment Interaction and Breast Cancer in Germany (Pesch et al., 2005; Justenhoven et al., 2008)	Germany	Incident breast cancer cases were enrolled from 2000 to 2004 from the Greater Bonn area (from all hospitals within the study region); cases were enrolled within 6 months of diagnosis.	Controls were selected from population registries from 31 communities in the greater Bonn area; matched to cases in 5-year age classes from 2001 to 2004.	23–80/24–80
MARIE: Mammary Carcinoma Risk Factor Investigation (Flesch-Janys et al., 2008)	Germany	Incident and prevalent cases diagnosed from 2001 to 2005 in the study region Hamburg in Northern Germany, and from 2002 to 2005 in the study region Rhein-Neckar-Karlsruhe in Southern Germany.	Two controls per case were randomly drawn from population registries and frequency matched by birth year and study region to the case. Controls were recruited from 2002 to 2006.	50–75/49–75
KBCP: Kuopio Breast Cancer Project (Hartikainen et al., 2005, 2006)	Finland	Women seen at Kuopio University Hospital between 1990 and 1995 because of breast lump, mammographic abnormality, or other breast symptom who were found to have breast cancer.	Age and long-term area-of-residence matched controls selected from the National Population Register and interviewed in parallel with the cases.	23–92/27–77
OBCS: Oulu Breast Cancer Study (Wedren et al., 2004; Erkko et al., 2007)	Finland	Consecutive incident cases diagnosed at the Oulu University Hospital between 2000 and 2004.	Healthy, consecutive, anonymous, female Finnish Red-Cross blood donors recruited in 2002 from the same geographical region in Northern Finland.	28–92/18–66
SASBAC: Singapore and Sweden Breast Cancer Study (Wedren et al., 2004)	Sweden	Incident cases from 1993 to 1995 identified via the 6 regional cancer registries in Sweden, to which reporting is mandatory.	Controls were randomly selected from the total population registry in 5-year age groups to match the expected age-frequency distribution among cases. Patients and controls were recruited from 1993 to 1995.	50–75/49–76

Blood-derived DNA samples of all cases and controls were genotyped for *UGT1A6*_19_T>G and *UGT1A6*_541_A>G by matrix-assisted laser desorption/ionization mass spectrometry (MALDI-TOF MS; Sequenom, San Diego, CA) as described previously (The MARIE-GENICA Consortium, 2010). Genetic analyses included negative controls and 10% randomly selected duplicate samples for quality control. Call rates were greater than 98% and repeated analyses of the duplicate samples showed 100% concordance. *UGT1A6*_19_T>G and *UGT1A6*_541_A>G genotype frequencies met the Hardy–Weinberg equilibrium.

Associations between *UGT1A6*_19_T>G and *UGT1A6*_541_A>G genotypes and breast cancer risk were analyzed by logistic regression adjusted for age and study using SPSS software v 15.0 (SPSS Inc., IBM Corporation, Somers, NY). Four subgroup analyses were performed including the analysis of ER-positive cases vs. controls, ER-negative cases vs. controls, PR-positive cases versus controls, and PR-negative cases vs. controls. Risk estimates were calculated as odds ratios (OR) with 95% confidence interval (95% CI) and corresponding *p*-value. Power calculation was done by nQuery Advisor® 4.0.

## Results

Our validation study had 80% power to detect a minimum OR of 1.06 and 1.07 for the *UGT1A6*_19_T>G and *UGT1A6*_541_A>G polymorphisms, respectively (α = 0.05, two-sided test). We observed no breast cancer risk association for the *UGT1A6*_541_A>G genotypes and none of the two variants showed an association with tumor ER and PR status (data not shown).

For the *UGT1A6*_19_T>G polymorphism, we confirmed an increased breast cancer risk for women carrying the homozygous variant GG genotype (Table 2). This finding complements our two-stage study in which carriers of the *UGT1A6*_19_GG genotype showed an OR of 1.17 (95% CI 1.03–1.34; *p* = 0.014) in the previous pilot investigation (stage 1) (The MARIE-GENICA Consortium, 2010). This result was based on adjustment for duration of any hormone replacement therapy, type of menopause, number of births, ever having breastfed, ever having smoked, number of mammograms, ever having had benign breast disease, family history of breast cancer in 1st degree relatives, body mass index, study region, and year of birth in 5 year categories. Adjustment for age and study only showed a similar risk effect with an OR of 1.09 (95% CI 1.02–1.16; *p* = 0.009) for the *UGT1A6*_19_GG genotype (Table 2). The confirmation of the increased overall breast cancer risk of OR 1.13 (95% CI 1.05–1.22; *p* = 0.001; Table 2) is reported herein (stage 2 using four independent cohorts BBCC, KBCP, OBCS, and SASBAC). The pooled analysis of BBCC, KBCP, OBCS, SASBAC, GENICA, and MARIE again confirmed the *UGT1A6*_19_GG genotype as a breast cancer risk factor with an OR of 1.09 (95% CI 1.04–1.14; *p* = 0.001; Table 2).

**Table 2:** **Association of *UGT1A6*_19_T>G with breast cancer risk**.

**Study**	***UGT1A6*_19_T>G genotypes and alleles**	**Cases (%)**	**Controls (%)**	***OR* (95% CI)**	***p*-value**
**PILOT STUDY**					
MARIE-GENICA	TT	1006 (32.0)	1882 (34.4)	1.00^a^	
(The MARIE-GENICA Consortium, 2010)	TG	1544 (49.2)	2644 (48.4)	1.10^b^ (0.99–1.21)	
	GG	589 (18.7)	940 (17.2)	1.17^b^ (1.03–1.34)	0.014^c^
	TT	1134 (32.3)	2017 (34.6)	1.00^a^	
	TG	1725 (49.1)	2807 (48.2)	1.10 (0.99–1.21)	0.051
	GG	654 (18.6)	1000 (17.2)	1.09 (1.02–1.16)	0.009
	TG+GG	2379	3807	1.12 (1.02–1.23)	0.013
	T	3556 (56.6)	6408 (58.6)	1.00^a^	
	G	2722 (43.4)	4524 (41.4)	1.08 (1.01–1.15)	0.012
**VALIDATION STUDY**					
BBCC, KBCP, OBCS, SASBAC	TT	1145 (31.5)	858 (32.4)	1.00^a^	
	TG	1643 (45.2)	1269 (47.9)	0.98^d^ (0.87–1.10)	0.686
	GG	847 (23.3)	521 (19.7)	1.13^d^ (1.05–1.22)	0.001
	TG + GG	2490	1790	1.06^d^ (0.95–1.19)	0.295
	T	3933 (54.1)	2987 (62.5)	1.00^a^	
	G	3337 (45.9)	1790 (37.5)	1.10 (1.02–1.18)	0.012
**POOLED ANALYSIS**					
MARIE-GENICA, BBCC, KBCP, OBCS, SASBAC	TT	2279 (31.9)	2875 (33.9)	1.00^a^	
	TG	3368 (47.1)	4076 (48.1)	1.03^d^ (0.96–1.11)	0.418
	GG	1501 (21.0)	1521 (18.0)	1.09^d^ (1.04–1.14)	0.001
	TG + GG	4869	5597	1.07^d^ (1.00–1.15)	0.041
	T	7926 (55.4)	9826 (58.0)	1.00^a^	
	G	6370 (44.6)	7118 (42.0)	1.11 (1.06–1.16)	5.86 × 10^−6^

CI, confidence interval; OR, odds ratio.

a Reference.

b OR adjusted for duration of any hormone replacement therapy, type of menopause, number of births, ever having breastfed, ever having smoked, number of mammograms, ever having had benign breast disease, family history of breast cancer in 1st degree relatives, and body mass index, study region and year of birth in 5 year categories.

c p-value from a log additive model as published.

d OR adjusted for age and study.

In addition to the genotype frequencies the risk effect of the *UGT1A6*_19_G allele was analyzed on the level of allele frequencies and showed ORs of 1.08 (95% CI 1.01–1.15: *p* = 0.012), 1.10 (95% CI 1.02–1.18; *p* = 0.12), and 1.11 (95% CI 1.06–1.16; *p* = 5.86 × 10^−6^) in the pilot and validation study as well as pooled analysis, respectively.

## Discussion

We confirmed the *UGT1A6*_19_GG genotype as an overall breast cancer risk factor, first, in an independent validation study including breast cancer cases and controls from Finland, Germany and Sweden as well as in a pooled analysis of more than 15,000 study subjects following our initial report from a German pilot study (The MARIE-GENICA Consortium, 2010). This finding supports the notion that reduced activity of detoxification enzymes such as UGT1A6 contribute to an overall breast cancer risk. The present study highlights the necessity to extend investigations for the discovery of breast cancer susceptibility loci to enzymes involved in detoxification processes despite their inherent problem of high homology which excludes them from standard GWAS approaches. As of yet, although GWAS approaches have delivered more than 20 common breast cancer risk loci (Turnbull et al., 2010; Ghoussaini et al., 2012), there is a possibility, that tailored approaches to cover variants in regions of sequence homology may add to the growing list of genetic risk factors.

It is of note that the herein observed ORs for the *UGT1A6*_19_G variant are in the range of the so far established common low-penetrance breast cancer susceptibility loci with ORs from 1.07 to 1.22 including polymorphisms in genes such as *FGFR2* and *TOX3*. As of yet, this set of more than 20 known susceptibility loci accounts for about 8% of the heritability of breast cancer (Ghoussaini et al., 2012). Therefore, it will be of particular interest to evaluate the *UGT1A6* polymorphism in concert with all known risk factors in large study collections to establish a more comprehensive breast cancer susceptibility panel.

We analyzed a potential association of the *UGT1A6*_19_T>G polymorphism with ER and PR status of breast tumors and observed no effect. Other histopathological variables were not included in the analyses herein due to missing data and variables as well as differences in the collection and documentation of these variables among studies. Since breast cancer is a heterogeneous disease it will be of interest to analyze the association of this polymorphism and breast cancer subtypes in large study collections with a broad set of hisopathological variables.

The role of a *UGT1A6* variant for breast cancer risk is reasonable because a reduction of enzyme activity has been previously suggested (Ciotti et al., 1997). Moreover, it is well known that UGT1A6 is involved in the conjugation of steroid hormones (Raftogianis et al., 2000) and increased levels of sex hormones are a known risk factor for breast cancer (Key et al., 2002). UGT1A6 also conjugates exogenous compounds which occur in the environment and in food, e.g., 4-ethylphenol or in drugs, e.g., acetaminophen (Harding et al., 1988; Bock and Kohle, 2005), which together with other UGT1A6 substrates have been suggested to affect breast cancer risk (Friis et al., 2008; Chen et al., 2011).

## Conclusion and perspectives

The present study which is limited to women of European descent holds the potential for further validation of the UGT1A6 and other functionally related enzymes in a more in-depth global context. Currently, we consider the prediction of breast cancer risk by polymorphisms of phase I and II enzymes in its initial stage and encourage further comprehensive studies to establish their contributory role in breast cancer risk. This should be done across populations and ethnicities as is potentially accessible through the Breast Cancer Association Consortium (BCAC) (Easton et al., 2007; Lambrechts et al., 2012). Notably, our finding expands the scope of known crucial pathways involved in breast cancer susceptibility from growth and cell cycle control to metabolic enzymes and confirms the link between the phase II enzyme UGT1A6 and breast cancer risk.

### Conflict of interest statement

The authors declare that the research was conducted in the absence of any commercial or financial relationships that could be construed as a potential conflict of interest.
